# Development of a nomogram using fundus photography to predict glaucoma progression in patients showing disc hemorrhage

**DOI:** 10.1038/s41598-020-71183-8

**Published:** 2020-09-04

**Authors:** Sangah Kim, Chan Keum Park, Eun Woo Kim, Sang Yeop Lee, Gong Je Seong, Chan Yun Kim, Hyoung Won Bae

**Affiliations:** 1 Siloam Eye Hospital, Seoul, South Korea; 2 Department of Ophthalmology, Severance Hospital, Institute of Vision Research, Yonsei University College of Medicine, 134 Sinchon-dong, Seodaemun-gu, Seoul, Republic of Korea; 3 Department of Ophthalmology, Maryknoll Hospital, Junggu, Busan, South Korea; 4 Department of Ophthalmology, Myung-Gok Eye Research Institute, Kim’s Eye Hospital, Konyang University College of Medicine, Seoul, South Korea

**Keywords:** Retina, Risk factors

## Abstract

To develop a nomogram to predict the progression of glaucoma by fundus photography in patients with disc hemorrhage. Retrospective review of the medical records of patients with disc hemorrhage, which was detected during follow up with open angle glaucoma, from January 2010 to March 2018. Patients were divided into glaucoma progression (n = 52) or non-progression (n = 38) groups. We assessed proximal location and morphology of disc hemorrhage; relationship to retinal nerve fiber layer defects with disc hemorrhage; and angular extent of disc hemorrhage, between groups using fundus photography. Multiple logistic regression analysis was performed to select prognostic factors, and we constructed a nomogram to predict glaucoma progression. The number of disc hemorrhage at the border of retinal nerve fiber layer defects (P = 0.001) and peripapillary disc hemorrhage (P = 0.008) were significantly higher in the progression group. We used angular extent; location of disc hemorrhage with retinal nerve fiber layer defects; and proximal location of disc hemorrhage to construct the nomogram. The area under the receiver operating characteristic curve of the nomogram was 0.847. We created the nomogram using fundus photography in patients showing disc hemorrhage as a novel and accurate screening method to predict glaucoma progression and aid clinicians to decide on the best treatment plan.

## Introduction

Disc hemorrhage (DH) is an important risk factor for the development and progression of glaucoma^1–3^; an Ocular Hypertension Treatment Study showed that DH was a risk factor for developing primary open angle glaucoma. In addition, according to the Collaborative Normal-Tension Glaucoma Study, DH was significantly associated with visual field (VF) progression^2^. However, not all patients with DH show progressive changes in glaucoma.

Previous studies have reported topographic features of DH and DH is often associated with rim nothcing at the bleeding site and predicts the location of retinal nerve fiber layer defects (RNFLD)^4–8^. Especially, many disc hemorrhages were located in the border between the localized RNFL defect and the relatively healthy-looking RNFL^9–11^. Nitta et al. found that RNFL defect enlarged in the direction towards DH and it may cause progression of glaucoma^11^. Also, the recent study found that a quadrant with DH had a faster glaucoma progression than a quadrant with non-DH^12^.

Fundus photography has been widely used in ophthalmology, including glaucoma assessments, because it is relatively inexpensive and easy to perform^13–17^. In addition, images can be saved and used for further clinical purposes at a later date, which is useful for monitoring disease progression. Therefore, fundus photography may be a useful tool to detect and observe DH in patients with glaucoma.

The purpose of this study was to develop a simple and novel tool for predicting the progression of glaucoma using the morphologic findings of DH from fundus photography. We created a nomogram based on a logistic regression model and sought to predict the probability of a particular outcome with a combination of important prognostic factors.

### Materials and methods

This study was a retrospective study conducted in the Ophthalmology Department of Yonsei University Severance Hospital. The Institutional Review Board (IRB) of Severance Hospital, Yonsei University College of Medicine (Seoul, Republic of Korea) reviewed and approved this study. Also, our approving IRB waived the need for us to obtain informed consent for study participations. All study conduct adhered to the tenets of the Declaration of Helsinki.

### Participants

The medical records of patients who visited our clinic between January 2010 and March 2016 were retrospectively reviewed. All patients underwent complete ophthalmologic examinations including slit-lamp biomicroscopy, best-corrected visual acuity (BCVA) measurement, dilated-fundus examination, intraocular pressure (IOP) measurement using Goldmann applanation tonometry, keratometry using an autokeratometer (RK-3, Canon, Lake Success, NY, USA), color disc photography (FF450 with Visupac, Carl Zeiss Meditec), red-free fundus photography (Carl Zeiss Meditec, Jena, Germany), and Cirrus OCT (Carl Zeiss Meditec). Axial length was measured using an IOL Master ocular biometric device (Carl Zeiss Meditec), and VF test (24-2 SITA Standard Algorithm, Humphrey Field Analyzer, Carl Zeiss Meditec, Dublin, CA, USA) was also performed for all patients.

Patients were included in the analyses if they (1) were diagnosed with open angle glaucoma (OAG); (2) DH had been detected at least once during follow up; (3) had been followed up for at least 3 years after DH documentation; (4) were aged between 18 and 70 years; (5) measured their vision with a Snellen chart and had a BCVA better than 20/40; (6) had a spherical refractive error between + 3.0 and − 6.0 diopters; and (7) had undergone ≥ 4 serial optical coherence tomography (OCT) and visual field (VF) examinations. We excluded patients with a history of surgical therapy, such as glaucoma filtering surgery. Patients with any other ocular disease that can cause disc hemorrhage, such as retinal vein occlusion, diabetic retinopathy, hypertensive retinopathy, ischemic optic neuropathy, papillitis, and posterior vitreous detachment were also excluded. Patients were excluded if high quality images could not be obtained, such as scans with signal strength less than 7, motion artifacts, and segmentation errors. If a patient had bilateral DH, one eye was randomly chosen as the study eye prior to analysis.

### Diagnosis of open angle glaucoma with disc hemorrhage

OAG was identified by two independent glaucoma specialists (SK and HWB) based on the typical appearance of a glaucomatous optic nerve head : cup/disc ratio > 0.7; cup/disc ratio asymmetry > 0.2; diffuse or focal neuroretinal rim thinning disc hemorrhage or vertical elongation of the optic cup, with corresponding glaucomatous VF defects and RNFLD using fundus photography. To confirm the open angle, gonioscopic examination was performed. VF, fundus photography, and red-free fundus photography were performed at intervals of 6 months. OCT examinations were performed at yearly intervals after the diagnosis of glaucoma. All DHs that occurred during the follow up period were analyzed based on fundus photography. DH was defined as an isolated blot or splinter hemorrhage on the optic disc or in the adjacent peripapillary area. All patients were evaluated for DH at every follow-up visit. Recurrent DH was defined as more than one DH during the total follow up period, with a DH event interval of more than 6 months. In recurrent DH, topographic features of fundus photo at the first DH was analyzed and applied to the nomogram.

### Topographic features of disc hemorrhage

All fundus photography and red free photography of DH were interpreted by two independent glaucoma specialists (SK and HWB). In cases of discrepancy, fundus photo and red free photo were reviewed by CYK.

**Figure 1:**
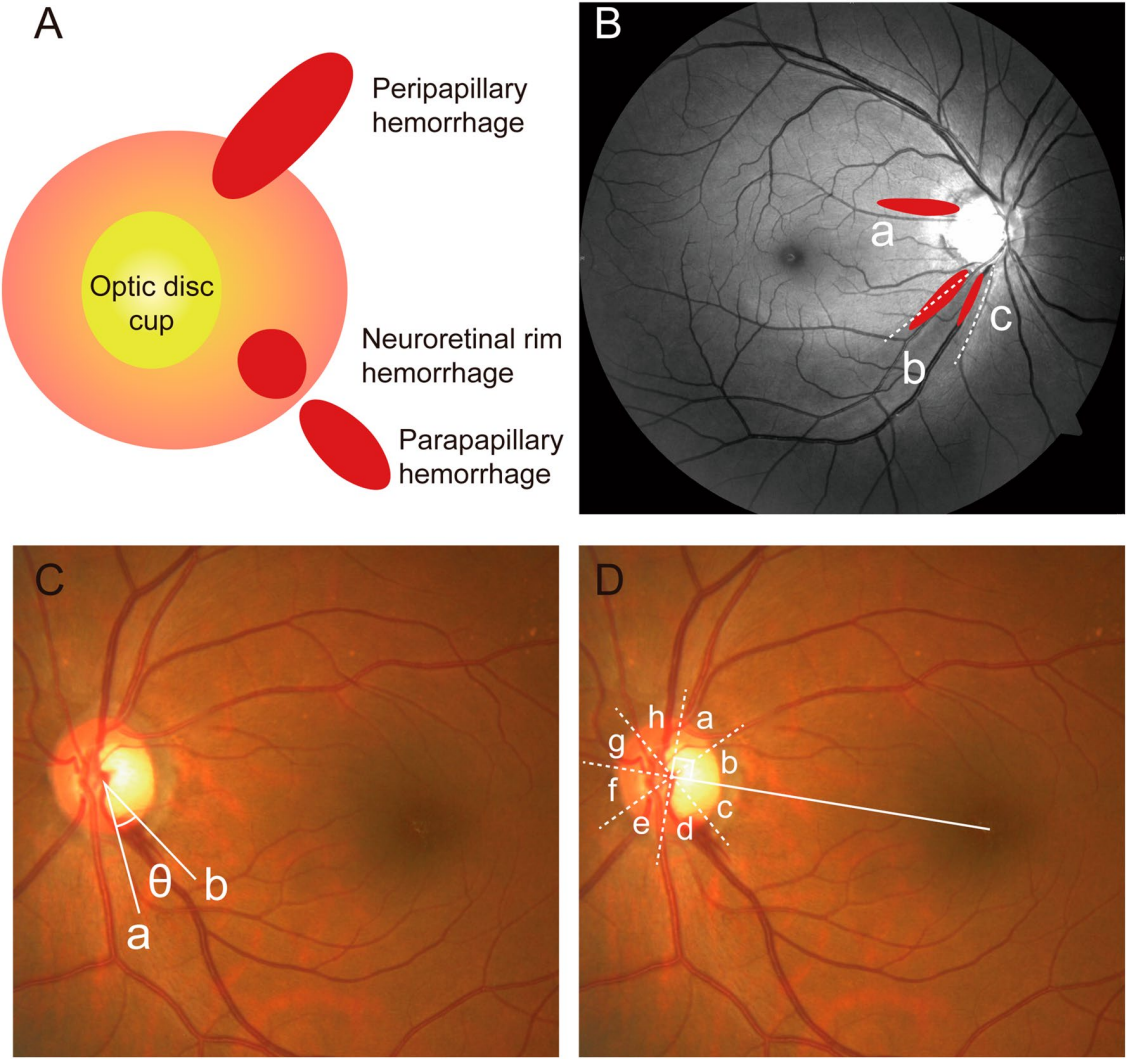
Example of the topographical analysis of DH in this study. (**A**) DH was divided into three types based on proximal location and morphology: (1) parapapillary hemorrhage located outside of the papilla; (2) located in the neuroretinal rim region; (3) peripapillary hemorrhage located in the neuroretinal rim and parapapillary region. (**B**) Red-free fundus photography to evaluate topographic relationship between DH and RNFLD. (a) DH at healthy RNFL; (b) DH at the border between RNFLD and healthy-looking RNFL; (c) DH in the region where RNFLD was present. (**C**) To measure the angular extent of DH, two lines (a and b) were drawn from the disc center to points at which the DH border met the disc. (**D**) To divide the position of DH by octant, we drew a reference line from the center of the optic disc to the center of the macula. We plotted a vertical line to the reference line and then drew two lines bisecting the divided quadrants to make eight quadrants. a, superotemporal-superior; b, superotemporal-inferior; c, inferotemporal-superior; d, inferotemporal-inferior; e, inferonasal-inferior; f, inferonasal-superior; g, superonasal-inferior; h, superonasal-superior. *DH* disc hemorrhage, *RNFL* retinal nerve fiber layer, *RNFLD* retinal nerve fiber layer defect.

DH was divided into three types based on proximal location and morphology (Fig. 1A)^18,19^: (1) parapapillary hemorrhage located outside of the papilla; (2) located in the neuroretinal rim region; or (3) peripapillary hemorrhage located both in the neuroretinal rim and parapapillary region. In this study, we recorded only one cup hemorrhage, which was inside the optic disc cup. This case was excluded to increase the diagnostic capacity of a nomogram.

To determine the topographic relationship between DH and retinal nerve fiber layer defects (RNFLDs), we divided the cases into 3 subtypes (Fig. 1B): (a) DH at the healthy retinal nerve fiber layer (RNFL); (b) DH at the border between RNFLD and healthy-looking RNFL; (c) DH within RNFLD.

To measure the angular extent of DH, two lines were drawn from the disc center to points at which the DH border met the disc margin (Fig. 1C). To divide the position of DH position by octant, we drew a reference line from the center of the optic disc to the center of the macula (Fig. 1D). Next, we plotted a vertical line to the reference line and then drew two lines bisecting the divided quadrants to make eight quadrants.

### Determination of glaucoma progression

We divided the patients with DH into two groups according to progression of glaucoma. The progression group were defined as those in which we observed and recorded the structural changes on fundus photographs and OCT, or functional changes on VF tests. The non-progression group was defined as those in which no progress was observed anywhere.

Structural progress was defined in two ways. The first progression of glaucoma was defined with changes in the glaucomatous optic disc from the fundus photo and the red-free photo, taken at the first and last clinic visit, (i.e., increase cup-to-disc ratio, change neuroretinal rim notching, adjacent vasculature position shift) which were independently evaluated by two glaucoma specialists (SK, HWB). Also changes in RNFL defect such as the appearance of a new RNFL defect or an increase in depth or width of an existing defect were noted. In cases of discrepancy, fundus photo and red free photo were reviewed by CYK. The second method of structural progression is a review of GCIPL and RNFL GPA event analysis using the average, superior, and inferior thickness summary parameters. We classified individuals as progression if they showed likely loss (red data points) on 2 consecutive visits in any GCIPL or RNFL summary parameter.

VF defect progression was detected by the event-based GPA that was obtained using the Humphrey VF Analyzer software. The GPA methodology was based on the Early Manifest Glaucoma Trial^20^. Briefly, the GPA established a baseline by averaging the results of the first 2 examinations, and successive follow-up examinations were compared to the baseline. Significant deterioration was recorded when *P* < 0.05. The presence of progression was considered when there was significant deterioration at the same 3 or more points on 2 consecutive tests. The date of detection of progression on GPA was adopted as the date of the progression event.

### Nomogram construction and validation

We performed univariate and multivariate logistic regression analyses to select significant risk factors for glaucoma progression. The estimated effect of each variable was ranked based on the estimated beta coefficient in multivariate logistic regression analysis. Points were serially assigned to other variables based on the absolute magnitude of the beta coefficients of the variables, and the relationship between each unit of beta coefficient and points was determined. A probability axis was then drawn based on the relationship between total points and the probability of glaucoma progression. Nomogram performance was quantified to determine discrimination and calibration. Discrimination performance was measured using the area under the receiver operating characteristic curve (AUC). Calibration was performed using graphical inspection of plots that compared observed outcome frequencies and predicted probabilities. The model was internally validated using the 1,000 bootstrapping method to obtain relatively unbiased estimates.

### Statistical analyses

Statistical analyses were performed using SPSS version 23.0 (IBM corporation, Armonk, NY, USA), SAS version 9.4 (SAS Institute, Cary, NC, USA), and R version 2.12.1 (The R Foundation for Statistical Computing, Vienna, Austria). Histogram analysis and the Shapiro–Wilk test were used to evaluate the normality of the data. Descriptive statistics were presented as mean ± standard deviation (SD) for normally distributed variables and median, first quartile, and third quartile for non-normally distributed variables. The independent *t*-test and Mann–Whitney *U* test were used to compare groups with normally and non-normally distributed data. Categorical data were compared using the χ^2^ test. Data were considered statistically significant when *P* < 0.05**.**

### Results

This study included data from 43 eyes of 43 OAG patients with DH who had detected glaucoma progression, and 35 eyes of 35 OAG patients with DH who had no detected glaucoma progression. The mean age was 60.0 years in the progression group and 63.5 years in the non-progression group. All patients used at least one glaucoma medication but there was no significant difference in glaucoma medication use and mean follow-up IOP. The baseline characteristics were similar between the groups (Table 1).

**Table 1:** Demographics and clinical characteristics of all study participants.

	Glaucoma with disc hemorrhage		*P*-value
	Progression group (n = 43 eyes)	Non-progression group (n = 35 eyes)	
Age (years)	60.0 (54.0, 67.7)	63.5 (56.0, 71.0)	0.371^†^
Male gender (%)	18 (41.9)	12 (34.3)	0.494
**Systemic comorbidity**			
DM (%)	6 (14.0)	9 (25.7)	0.190
HTN (%)	12 (27.9)	10 (28.6)	0.948
Use of ASA (%)	5 (11.6)	6 (17.1)	0.486
Number of glaucoma medication use	1.0 (1.0, 2.0)	1.0 (1.0, 2.0)	0.115^†^
Mean follow-up IOP (mmHg)	13.05 ± 2.08	13.61 ± 2.00	0.236*
Spherical equivalent (D)	− 0.62 (− 4.00, − 0.25)	− 0.90 (− 4.25, 0.75)	0.868^†^
Axial length (mm)	24.57 ± 1.71	24.83 ± 0.95	0.699*
Central corneal thickness (μm)	528.5 (494.5, 551.5)	530.5 (517.75, 549.25)	0.865^†^
Baseline mean RNFL thickness (μm)	81.09 ± 11.86	82.94 ± 9.60	0.657*
Baseline mean GCIPL thickness (μm)	76.5 (69.0, 80.0)	77.0 (72.75, 81.0)	0.888^†^
Baseline MD of VF (dB)	− 3.35 (− 6.21, − 1.55)	− 2.80 (− 4.86, − 1.35)	0.755^†^
Rate of average RNFL thinning (μm/year)	− 1.09 ± 0.99	− 0.15 ± 1.27	0.001*
Rate of superior RNFL thinning (μm/year)	− 0.84 ± 1.86	− 0.22 ± 1.54	0.145*
Rate of inferior RNFL thinning (μm/year)	− 1.74 ± 1.70	− 0.09 ± 2.94	0.005*
Rate of average GCIPL thinning (μm/year)	− 0.92 (− 1.23, − 0.65)	− 0.48 (− 0.66, − 0.03)	< 0.001^†^
Rate of superior GCIPL thinning (μm/year)	− 0.41 (− 0.78, − 0.09)	− 0.42 (− 0.52, − 0.04)	0.299^†^
Rate of inferior GCIPL thinning (μm/year)	− 1.14 (− 1.78, − 0.77)	− 0.67 (− 0.84, − 0.27)	< 0.001^†^
MD slope (dB/year)	− 0.05 (− 0.35, 0.48)	0.23 (− 0.07, 0.56)	0.204^†^
Follow-up duration (month)	63.30 ± 17.78	60.34 ± 17.54	0.464*

Data are presented as a mean ± standard deviation and a median (first, third quartiles).

Data are presented as the number of patients (%) for categorical variables.

Comparisons were performed using the independent t-test and the Mann–Whitney U-test for continuous variables.

Comparisons were performed using the chi-square test for categorical variables.

*DM* diabetes mellitus, *HTN* hypertension, *ASA* acetylsalicylic acid, *IOP* intraocular pressure, *D* diopter, *RNFL* retinal nerve fiber layer, *GCIPL* ganglion cell and inner plexiform layer, *MD* mean deviation, *VF* visual field, *dB* decibel, *PSD* pattern standard deviation.

P, comparison between two groups.

*Independent t-test.

^†^Mann–Whitney U-test.

Regarding the characteristics of DH, there were no significant differences in recurrence rate, bilaterality, duration of DH, angular extent of DH, and octant location of DH between the groups (Table 2). However, there was a significant difference in the proximal location of DH (*P* = 0.008). The most common proximal location of DH was the peripapillary (58.1%) and parapapillary (54.2%) region in the progression and non-progression groups, respectively. In terms of the positional relationship between DH and RNFLD, DH at the border of RNFLD was observed in 81.4% and 37.1% and DH at a location without RNFLD found in 6.9% and 45.7% of eyes in the progression and non-progression group, respectively.

**Table 2:** Topographic features and characteristics of dis hemorrhage.

	Glaucoma with disc hemorrhage		*P*-value
	Progression group (n = 43 eyes)	Non-progression group (n = 35 eyes)	
DH recurrence (%)	7 (16.3%)	8 (22.97%)	0.463
Bilaterality of DH (%)	8 (18.6%)	2 (5.7%)	0.090
Angular extent of DH (degrees)	10.37 (6.88, 15.37)	12.14 (7.40, 15.06)	0.234
**Proximal location of DH (%)**			0.008
Parapapillary	9 (20.9%)	19 (54.2%)	
Peripapillary	25 (58.1%)	13 (37.2%)	
Neuroretinal rim	9 (20.9%)	3 (8.5%)	
**DH location with RNFLD (%)**			0.001
Without RNFLD	3 (6.9%)	16 (45.7%)	
Within RNFLD	5 (11.6%)	6 (17.1%)	
Border of RNFLD	35 (81.4%)	13 (37.1%)	
**Octant location of DH (%)**			0.355
Superotemporal-superior	9 (20.9%)	10 (28.6%)	
Superotemporal-inferior	1 (2.3%)	3 (8.6%)	
Inferotemporal-superior	7 (16.3%)	5 (14.3%)	
Inferotemporal-inferior	25 (58.1%)	14 (40.0%)	
Inferonasal-inferior	0 (0.0%)	1 (2.9%)	
Inferonasal-superior	0 (0.0%)	1 (2.9%)	
Superonasal-inferior	1 (2.3%)	0 (0.0%)	
Superonasal-superior	0 (0.0%)	1 (2.9%)	

Data are presented as a median (first, third quartiles).

Data are presented as the number of patients (%) for categorical variables.

Comparisons were performed using the Mann–Whitney U-test for continuous variables.

Comparisons were performed using the chi-square test for categorical variables.

*DH* disc hemorrhage, *RNFLD* retinal nerve fiber layer defect.

P, comparison between two groups.

**Figure 2:**
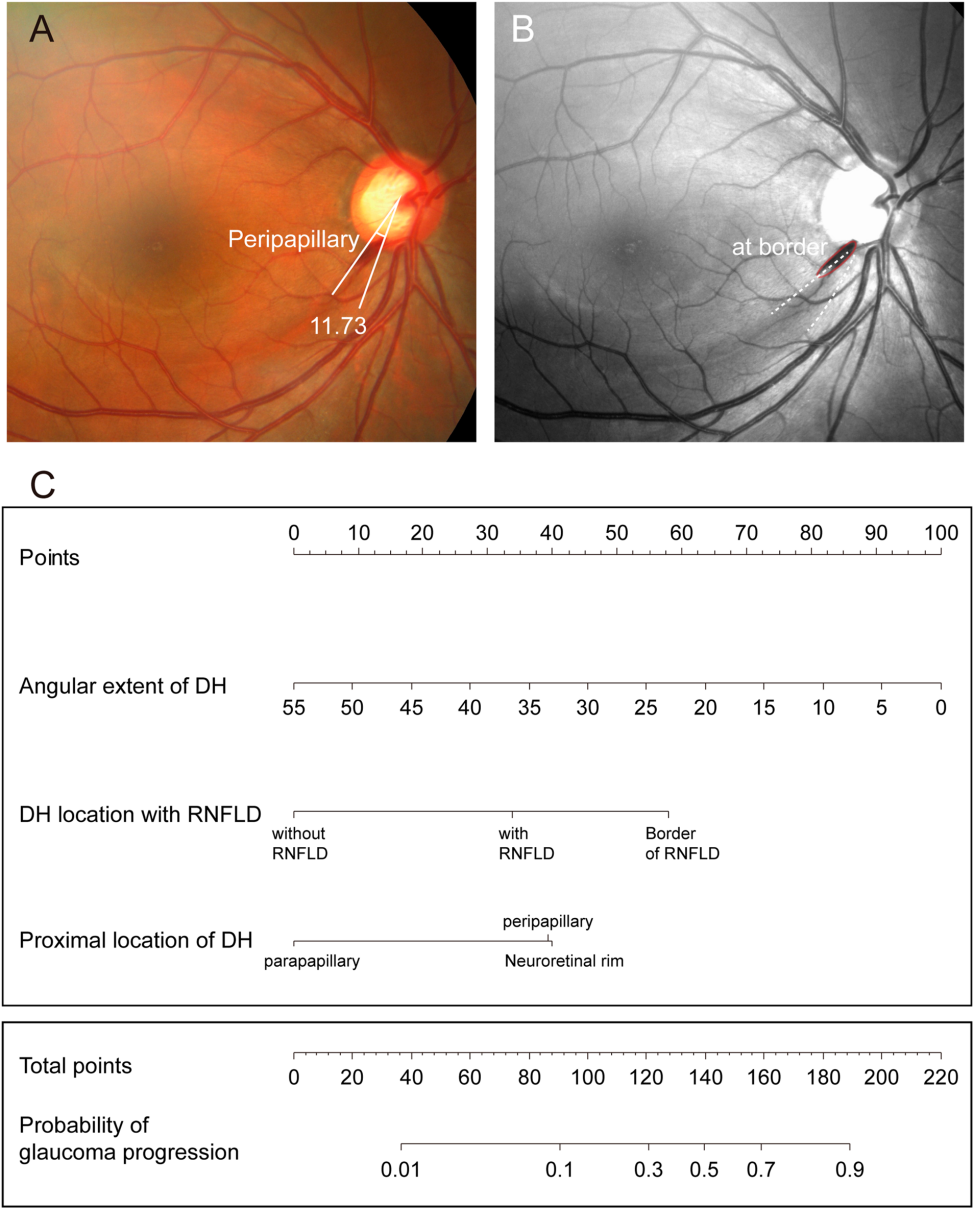
Example of the nomogram and practical usage. (**A**) A 46-year-old man presented with DH and revealed glaucoma progression. He was diagnosed with peripapillary DH with an angular extent of 11.73°. (**B**) DH located at the border of RNFLD. (**C**) For predicting the probability of glaucoma progression in an individual patient, parameter values (DH location with RNFLD, proximal location of DH, and angular extent of DH) are first placed on the corresponding variable axes. Starting from these points along the axes, vertical lines are drawn upward towards the point scale in the upper-most corner of the nomogram to determine the number of points that is to be assigned to each parameter. For example, DH at the border of RNFLD would correspond to about 58 points (arrow). Peripapillary DH and angular extent would be 39 and 80 points, respectively (arrow). Summing these points gives a total of 177 points. Lastly, a vertical line is drawn downward towards the scale along the bottom to obtain the probability of glaucoma progression. In this example, 177 points would correspond to a probability of approximately > 0.8 (arrow). *DH* disc hemorrhage, *RNFLD* retinal nerve fiber layer defect.

**Figure 3:**
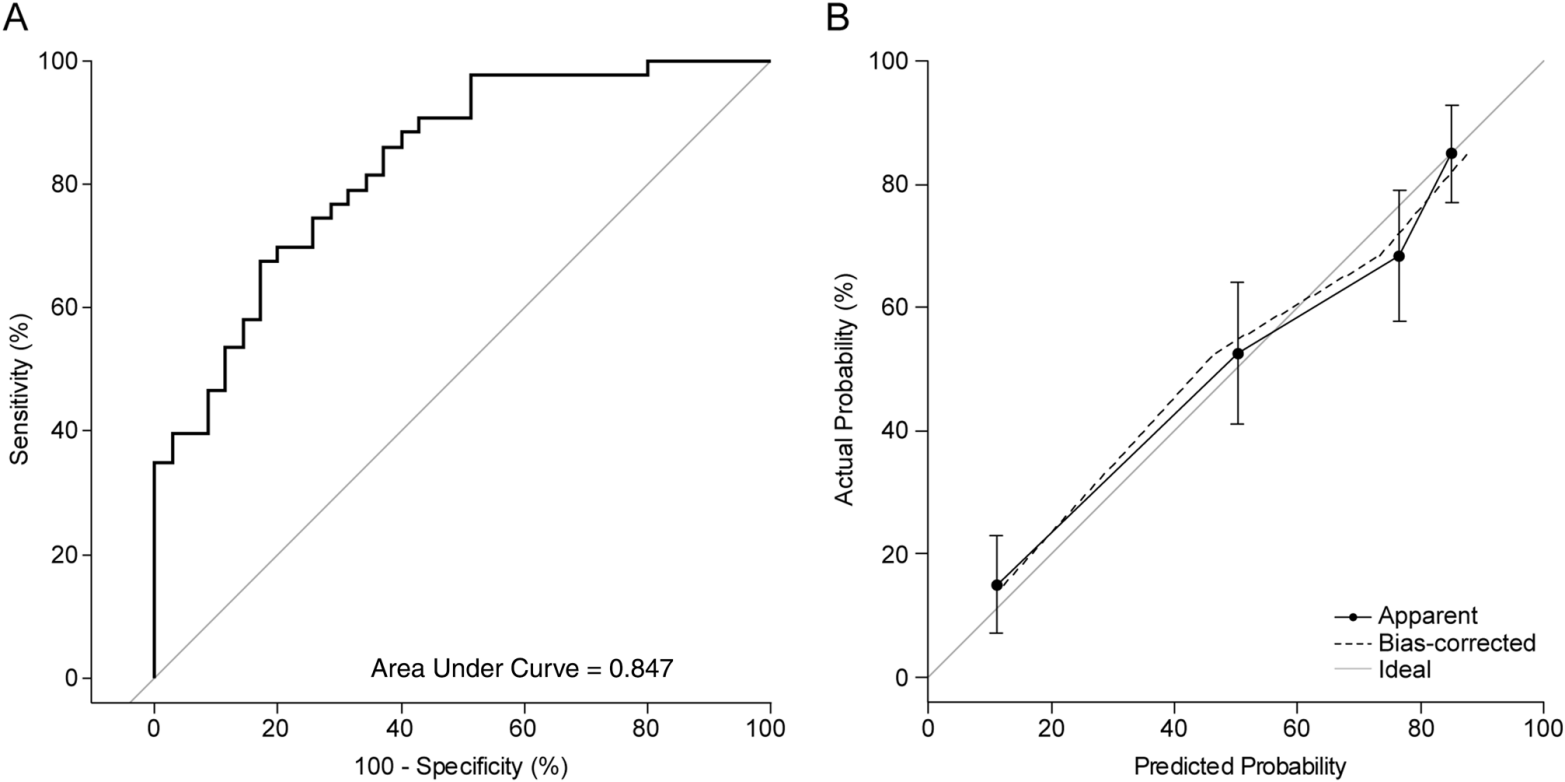
(**A**) Internal validation of the nomogram to predict glaucoma progression in DH patients. Area under the receiver operating characteristic curve is 0.847. (**B**) Calibration plot of the logistic model. Dotted line indicates location of the ideal nomogram, in which predicted and actual probabilities are identical. *DH* disc hemorrhage.

**Figure 4:**
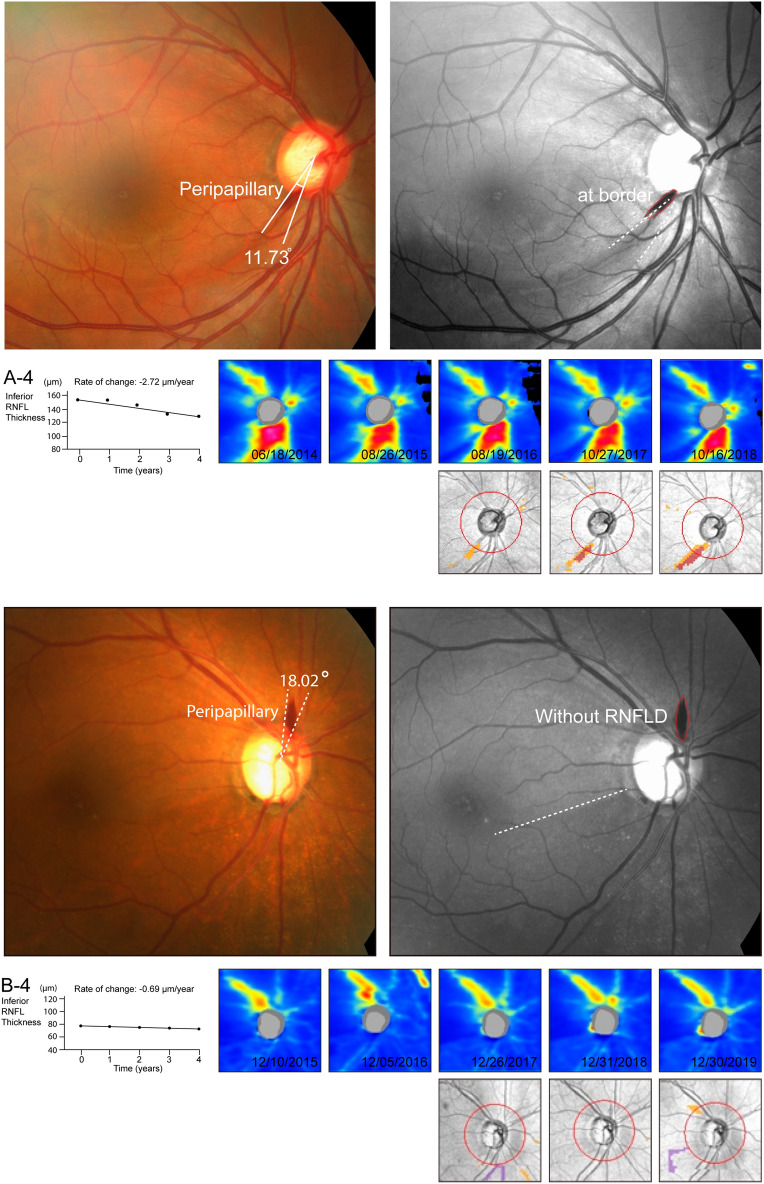
The results of ophthalmological evaluation and findings of representative cases. (**A**) A 46-year-old man presented with DH. (**A-1**) He was diagnosed with peripapillary hemorrhage and the angular extent was 11.73°. (**A-2**) DH located at the border of RNFLD. (**A-3**) Progressive RNFL thinning is demonstrated in the serial illustration of a sectorial RNFL thickness map. (**A-4**) The rate of inferior RNFL thinning was − 2.72 μm year^−1^. (**B**) A 62-year-old woman presented with DH. (**B-1**) She was diagnosed with peripapillary hemorrhage and the angular extent was 18.02°. (**B-2**) DH located at healthy-looking RNFL. (**B-3**) Non-progressive RNFL thinning is demonstrated in the serial illustration of sectorial RNFL thickness map. (**B-4**) The rate of inferior RNFL thinning was + 1.75 μm year^−1^. According to the nomogram, the total scores were 177 and 104 for patients A and B. The probability of glaucoma progression was > 0.8 and < 0.2 for patients A and B*. DH* disc hemorrhage, *RNFLD* retinal nerve fiber layer defect, *RNFL* retinal nerve fiber.

Univariate logistic regression analysis showed that the proximal location of DH and DH location with RNFLD were significantly associated with glaucoma progression (Table 3). The odds of glaucoma progression in DH at the neuroretinal rim were six times higher than the odds of parapapillary hemorrhage. In addition, the odds of DH at the RNFLD border were fourteen times higher than the odds of DH in the healthy-looking RNFL. In the multivariate logistic regression analysis, proximal location of DH and DH location with RNFLD were significantly associated with the glaucoma progression. Based on the multivariate logistic regression analyses, we construct a nomogram to predict the progression of glaucoma (Fig. 2C). The AUC of the model was 0.847 (Fig. 3A). The calibration curve revealed that the bias-corrected lines, which represent the performance of the bootstrap-corrected nomogram, were close to an ideal line, indicating good predictive value. Serial results of ophthalmological evaluations of the representative cases are shown in Fig. 4.

**Table 3:** Univariate and multivariate logistic regression analysis of factors predicting glaucoma progression.

	Univariate		Multivariate	
	OR (95% CI)	P-value	OR (95% CI)	P-value
Angular extent of DH	0.955 (0.900, 1.014)	0.134	0.922 (0.837. 1.016)	0.100
**Proximal location of DH**				
Parapapillary	Ref		Ref	
Peripapillary	4.060 (1.437, 11.467)	0.008	5.907 (1.498, 23.288)	0.011
Neuroretinal rim	6.333 (1.373. 29.205)	0.017	5.758 (1.032, 32.123)	0.045
**DH location with RNFLD**				
Without RNFLD	Ref		Ref	
Within RNFLD	4.444 (0.803, 24.609)	0.087	4.502 (0.663, 30.576)	0.123
Border of RNFLD	14.359 (3.585, 57.519)	< 0.001	13.202 (2.975, 58.586)	< 0.001

*OR* odds ratio, *CI* confidence interval, *IOP* intraocular pressure, *DM* diabetes mellitus, *MD* mean deviation, *VF* visual field, *RNFL* retinal nerve fiber layer, *GCIPL* ganglion cell and inner plexiform layer, *DH* disc hemorrhage.

## Discussion

The present study demonstrated that the angular extent of DH, proximal location of DH, and location of DH with RNFLD are reliable predictors for glaucoma progression with DH patient. With these predictors, we constructed a nomogram to predict the risk of glaucoma progression, which we found was well calibrated. To the best of our knowledge, this is the first study to compare progression and non-progression of glaucoma according to morphologic features of DH using fundus photography.

There are two theories to explain DH occurrence. The first is the vascular hypothesis, which suggests that vascular dysregulation and systemic factors, such as hypertension, hypotension, diabetes mellitus, and systemic medication (including acetylsalicylic acid), can induce ischemic changes in the optic disc^21–24^. The second theory is the mechanical hypothesis, which suggests that microvascular disruption around the optic disc, such as lamina cribrosa or RNFL levels, induce DH^11,24,25^. Both theories could explain the occurrence of DH; however, the mechanical theory is more likely to correlate DH with glaucoma progression. Nitta et al. found that RNFLD enlarges in the direction towards DH. They have hypothesized that RNFLD enlargement induces DH via disruption of the capillary network around the border of RNFLD^11^. These processes also lead to the progression of VF. This is a possible cause for the high incidence of DH at the border of RNFLD in the progression group in this study.

This study found that DH at the neuroretinal rim and the extent of angular narrowing in DH appeared to be associated with glaucoma progression. Kim et al. have reported that the angular extent of DH in a normal tension glaucoma group was smaller than patients with high tension glaucoma^26^. However, there was no difference in IOP between the groups in this study. One possible reason for the proximal location of DH is that DH outside the optic disc may be more likely to be caused by a factor unassociated with glaucoma. Furthermore, if the angle of DH is wide, there is a higher likelihood of bleeding on the more superficial layer; bleeding related to posterior vitreous detachment; or bleeding due to anti-platelet administration. Therefore, additional follow-up studies are needed to clarify these mechanisms.

In the present study, DH recurrence rate was not different between the groups. Many studies have shown that the recurrence of DH is associated with glaucoma progression; however, there are conflicting data. Ishida et al. found that 71.9% of patients had recurrent hemorrhage, and recurrent DH was significantly more progressive in VF than non-recurrent DH^27^. However, de Beaufort et al. found no significantly difference in the rate of VF progression between groups^28^. Kim et al. did not find a difference in the rate of VF progression, but showed that recurrent DH was significantly greater than single DH with respect to optic disc deteriotation^29^. These conflicting results can be explained by differences in the duration of follow-up and differences in the interval between observations.

Fundus photography is simple to perform, cheap, and can provide a lot of information; therefore, it is widely used as an examination in regular check-ups. In addition, images can be saved and used for further assessments, which is useful in the detection and observation of DH. Therefore, a nomogram with fundus photography is simple and cost-effective screening method to predict glaucoma progression in DH patients.

This study has several limitations. First, this was a retrospective study; thus, we could not control for selection bias. In addition, although the POAG and DH incidence rates vary by race, this study was conducted on a single race, thus having limits to the study’s generalization^30–32^. We have tried to control these biases, this nomogram should be used for general pupulations considering these limitations. Second, we had a relatively small number of cases, and the nomogram may have limited predictive power. However, we performed internal validation, which revealed good predictive power. Third, all patients were consistently observed every six months. It was possible that this did not detect all patients with DH. Lastly, it remains unclear whether the angular extent and proximal location of DH represent a pathological causal relationship in the progression of glaucoma. Nonetheless, this is the first study to predict glaucoma progression using the morphologic findings of DH with easy-to-access fundus photography in a clinical setting.

In conclusion, we developed a nomogram as a novel and accurate screening method to predict glaucoma progression in patients with DH. This study is the beginning of efforts to subdivide DH according to morphologic features and the nomogram will assist ophthalmologists with patient care decisions for those at higher risk for glaucoma progression.
